# Supplementary material for: Empirical methods for controlling false positives and estimating confidence in ChIP-Seq peaks
Source: BMC Bioinformatics. 2008 Dec 5;9:523. doi: 10.1186/1471-2105-9-523 (PMC2628906; doi:10.1186/1471-2105-9-523)
Supplement: Additional File 1 — A variety of html documents from the USeq web site detailing the available applications, their best usage, output file type descriptions, command line menus, etc. [file 1471-2105-9-523-S1.zip › USeqUserGuides/JavaDoc.zip!/JavaDoc/edu/utah/seq/analysis/EnrichedRegionMaker.html]

EnrichedRegionMaker


|  |  |  |
| --- | --- | --- |
| **Overview** \| **Package** \| **Class** \| **Use** \| **Tree** \| **Deprecated** \| **Index** \| **Help** | |  |
| **PREV CLASS**   **NEXT CLASS** | **FRAMES**    **NO FRAMES**     **All Classes** |  |
| SUMMARY: NESTED \| FIELD \| CONSTR \| METHOD | DETAIL: FIELD \| CONSTR \| METHOD |  |


---


## edu.utah.seq.analysis Class EnrichedRegionMaker

```
java.lang.Object
  edu.utah.seq.analysis.EnrichedRegionMaker
```

---

``` public class EnrichedRegionMaker extends java.lang.Object ```

Collapses an array of window into and array of enriched regions based on score and max gap.

---

**Constructor Summary**

`EnrichedRegionMaker(int maxGap,
int scoreIndex,
SmoothingWindowInfo[] windowInfo)`

`EnrichedRegionMaker(java.lang.String[] args)`


| **Method Summary** | |
| --- | --- |
| `void` | `addIt()` |
| `boolean` | `checkScore(SmoothingWindow win)` |
| `int` | `countEnrichedRegions(boolean setStrippedWindows, float threshold)`             Returns the number of ERs that would have been made. |
| `SmoothingWindow` | `fetchGoodWindow()` |
| `void` | `filterWindows()` |
| `static void` | `main(java.lang.String[] args)` |
| `void` | `makeEnrichedRegions()`             Makes ERs given a window array. |
| `void` | `makeSetNumberEnrichedRegions()`             Will attempt to make the desired number of ERs |
| `void` | `pickPeaks()`             Attempts to pick the best peak within each enriched region. |
| `static void` | `printDocs()` |
| `static void` | `printEGRFile(EnrichedRegion[] ers, java.io.File egr, java.lang.String genomeVersion, java.lang.String[] scoreLabels)`             Writes out an egr file, used by IGB for graph display. |
| `static void` | `printEGRFileForBestSubWindow(EnrichedRegion[] ers, java.io.File egr, java.lang.String genomeVersion)`             Writes out an egr file for best sub windows, used by IGB for graph display. |
| `static void` | `printEnrichedRegionReport(EnrichedRegion[] ers, java.io.File spreadSheetFile, java.lang.String genomeVersion, java.lang.String[] scoreDescriptions)`             Writes out an excel compatible tab delimited spreadsheet with a hyperlink to IGB. |
| `static void` | `printGFFFile(EnrichedRegion[] ers, java.io.File egr, java.lang.String genomeVersion, int index, java.lang.String[] scoreLabels)`             Prints a simple gff file, the index is used to pick a score to place in the score column. |
| `static void` | `printGFFFileForBestSubWindow(EnrichedRegion[] ers, java.io.File egr, java.lang.String genomeVersion)`             Prints a simple gff file, the index is used to pick a score to place in the score column. |
| `void` | `printSubWindowGraphFiles(EnrichedRegion[] ers, java.io.File saveDir, java.lang.String genomeVersion)` |
| `void` | `processArgs(java.lang.String[] args)`             This method will process each argument and assign new varibles |
| `boolean` | `writeEnrichedRegions(java.io.File swiFile)`             Writes out ER reports. |
| `void` | `writeSubWindowBarFiles(java.io.File saveDir, java.lang.String genomeVersion)`             Writes the sub windows for each enriched region, note also sorts enriched regions by position |

**Methods inherited from class java.lang.Object**

`equals, getClass, hashCode, notify, notifyAll, toString, wait, wait, wait`

**Constructor Detail**

### EnrichedRegionMaker

```
public EnrichedRegionMaker(int maxGap,
                           int scoreIndex,
                           SmoothingWindowInfo[] windowInfo)
```

---


### EnrichedRegionMaker

```
public EnrichedRegionMaker(java.lang.String[] args)
```


**Method Detail**

### pickPeaks

```
public void pickPeaks()
```

:   Attempts to pick the best peak within each enriched region.

---


### writeEnrichedRegions

```
public boolean writeEnrichedRegions(java.io.File swiFile)
```

:   Writes out ER reports.

---


### printSubWindowGraphFiles

```
public void printSubWindowGraphFiles(EnrichedRegion[] ers,
                                     java.io.File saveDir,
                                     java.lang.String genomeVersion)
```

---


### writeSubWindowBarFiles

```
public void writeSubWindowBarFiles(java.io.File saveDir,
                                   java.lang.String genomeVersion)
```

:   Writes the sub windows for each enriched region, note also sorts enriched regions by position

---


### printGFFFileForBestSubWindow

```
public static void printGFFFileForBestSubWindow(EnrichedRegion[] ers,
                                                java.io.File egr,
                                                java.lang.String genomeVersion)
```

:   Prints a simple gff file, the index is used to pick a score to place in the score column.

---


### printEGRFileForBestSubWindow

```
public static void printEGRFileForBestSubWindow(EnrichedRegion[] ers,
                                                java.io.File egr,
                                                java.lang.String genomeVersion)
```

:   Writes out an egr file for best sub windows, used by IGB for graph display.

---


### printEGRFile

```
public static void printEGRFile(EnrichedRegion[] ers,
                                java.io.File egr,
                                java.lang.String genomeVersion,
                                java.lang.String[] scoreLabels)
```

:   Writes out an egr file, used by IGB for graph display.

---


### printGFFFile

```
public static void printGFFFile(EnrichedRegion[] ers,
                                java.io.File egr,
                                java.lang.String genomeVersion,
                                int index,
                                java.lang.String[] scoreLabels)
```

:   Prints a simple gff file, the index is used to pick a score to place in the score column.

---


### printEnrichedRegionReport

```
public static void printEnrichedRegionReport(EnrichedRegion[] ers,
                                             java.io.File spreadSheetFile,
                                             java.lang.String genomeVersion,
                                             java.lang.String[] scoreDescriptions)
```

:   Writes out an excel compatible tab delimited spreadsheet with a hyperlink to IGB.

---


### makeSetNumberEnrichedRegions

```
public void makeSetNumberEnrichedRegions()
```

:   Will attempt to make the desired number of ERs

---


### makeEnrichedRegions

```
public void makeEnrichedRegions()
```

:   Makes ERs given a window array.

---


### countEnrichedRegions

```
public int countEnrichedRegions(boolean setStrippedWindows,
                                float threshold)
```

:   Returns the number of ERs that would have been made.
    If setStrippedWindows, will set the stripped SmoothingWindows in the SmoothingWindowInfo at each call
    this should be much faster with increasing stringent threshold calls.
    Assumes SmoothingWindows are sorted by position, smallest to largest

---


### addIt

```
public void addIt()
```

---


### fetchGoodWindow

```
public SmoothingWindow fetchGoodWindow()
```

---


### checkScore

```
public boolean checkScore(SmoothingWindow win)
```

---


### main

```
public static void main(java.lang.String[] args)
```

---


### processArgs

```
public void processArgs(java.lang.String[] args)
```

:   This method will process each argument and assign new varibles

---


### printDocs

```
public static void printDocs()
```

---


### filterWindows

```
public void filterWindows()
```


---


|  |  |  |
| --- | --- | --- |
| **Overview** \| **Package** \| **Class** \| **Use** \| **Tree** \| **Deprecated** \| **Index** \| **Help** | |  |
| **PREV CLASS**   **NEXT CLASS** | **FRAMES**    **NO FRAMES**     **All Classes** |  |
| SUMMARY: NESTED \| FIELD \| CONSTR \| METHOD | DETAIL: FIELD \| CONSTR \| METHOD |  |


---
